# Biological and environmental datasets from the August 2017 total solar eclipse

**DOI:** 10.1016/j.dib.2018.10.008

**Published:** 2018-10-05

**Authors:** Emma M. Brinley Buckley, Andrew J. Caven, Benjamin L. Gottesman, Mary J. Harner, Bryan C. Pijanowski, Michael L. Forsberg

**Affiliations:** a Department of Communication, University of Nebraska at Kearney, Kearney, NE 68849, USA; b The Crane Trust, Wood River, NE 68883, USA; c Department of Forestry and Natural Resources, Purdue University, West Lafayette, IN 47907, USA; d Center for Global Soundscapes, Purdue University, West Lafayette, IN 47907, USA; e Department of Biology, University of Nebraska at Kearney, Kearney, NE 68849, USA; f Department of Agricultural Leadership, Education & Communication, University of Nebraska, Lincoln 68583, USA; g Michael Forsberg Photography, Lincoln, NE 68542, USA

## Abstract

The datasets in this article are associated with the research article ‘*Assessing biological and environmental effects of a total solar eclipse with passive multimodal technologies’* (Brinley Buckley et al., 2018). We documented biotic and abiotic changes during a total solar eclipse on 21 August 2017, in south-central Nebraska, USA, with a multimodal suite of tools, including time-lapse camera systems, data loggers, and sound recording devices. Time-lapse images were used to approximate changes in light, data loggers were used to record temperature and humidity, and sound recordings were used to calculate acoustic indices characterizing variation in the soundscape, as well as to manually identify and estimate avian vocalization activity.

**Specifications table**

**Table:** 

Subject area	*Ecology*
More specific subject area	*Total solar eclipse, soundscape, image-analysis, animal response, passive monitoring*
Type of data	*Light measurements, acoustic indices, avian activity, temperature, humidity*
How data was acquired	*Time-lapse camera systems, acoustic recording devices, data-loggers*
Data format	*Excel file, raw, analyzed*
Experimental factors	*Data were collected at several study locations before, during, and after a total solar eclipse*
Experimental features	*Continuous passive monitoring of visual, sonic, and atmospheric changes.*
Data source location	*Central Platte River Valley, Nebraska, USA*
Data accessibility	*Available in*Transparency document, Appendix A*as an Excel file*
Related research article	*Brinley Buckley et al. 2018. Assessing biological and environmental effects of a total solar eclipse with passive multimodal technologies. Ecological Indicators*

**Value of the data**

• Data document biological and environmental changes during a total solar eclipse, a natural phenomenon with limited empirical and quantitative documentation of biotic and environmental changes.
• The combination of time-lapse imagery, sound recordings, and environmental data demonstrates how multimodal remote sensing toolkits can effectively be used to characterize complex responses to environmental change.
• Data can be used to study changes in photic conditions and how animals reacted to rapid environmental changes.

## 1 Data

The Supplementary material ‘SolarEclipse_databrief.xlxs’ contains three datasheets: “Environmental,” “Acoustic Indices,” and “Avian Activity.” In all datasheets, Time is reported as Central Daylight Time (CDT), and Site is the sampling location in the central Platte River Valley, Nebraska.

### 1.1 Environmental

Environmental variables recorded during a total solar eclipse at two sites. Light value (LV) is a proxy for sunlight illumination on the landscape. Temperature is in degrees Celsius. Relative humidity is a percentage. These variables were collected at one-minute intervals.

### 1.2 Acoustic indices

Values obtained by analyzing sound recordings with three acoustic indices at four sites; RMS is the Root Mean Squared amplitude, ACI is the Acoustic Complexity Index, and ASA is the Average Signal Amplitude. RMS was calculated over the full frequency bandwidth of the recordings (0–8 kHz), while ACI and ASA were calculated on sounds between 2 and 8 kHz. The 5-h recordings from each of the four study sites were analyzed at 2.5-min intervals to match the duration of totality during the total solar eclipse.

### 1.3 Avian activity

Vocalization activity of all bird species detected during specified time intervals at four sites. Species is name of bird and is denoted by four letter alpha codes for standardized common names of birds (see Fig 6. in [1] for more details). Five time intervals of the eclipse were analyzed. Pre-eclipse represents pre-eclipse (30-min before eclipse began, 11:01:30–11:04:00); 95a is start of > 95% eclipsed (12:51:00–12:53:30); Totality is 100% obscuration (12:58:30–13:01:00); 95b is end of > 95% eclipsed (13:06:00–13:08:30); and Post-eclipse is 30-min after eclipse ended (14:56:00–14:58:30).

## 2 Experimental design, materials, and methods

Datasets were collected in the Central Platte River Valley of Nebraska, USA, during a total solar eclipse on 21 August 2017. The eclipse lasted for 2 hr and 52 min, from 11:34 h to 14:26 h (Central Daylight Time; CDT), and totality occurred for approximately 2 min and 34 sec between 12:58 h and 13:01 h CDT. A multimodal suite of tools were installed across three locations: Mormon Island of the Crane Trust (2025 ha; 40.798306 °N, − 98.416298 °W; 581 m elev.), Beaver Lodge (40.781636°N, − 98.472850°W; 520 m elev.) and Trout Pond (40.787715°N, − 98.464154°W; 520 m elev.) at Shoemaker Island of the Crane Trust, and Audubon׳s Rowe Sanctuary (980 ha; 40.669323°N, − 98.887926°W, 633 m elev.).

Data loggers and time-lapse cameras were deployed at two locations, Rowe Sanctuary and Mormon Island, to document changes in temperature, humidity, and sunlight at 30 s intervals. Both the cameras, Nikon D300 cameras (Exposure compensation: − 0.3, ISO: 400; aperture-priority: Rowe Sanctuary- f/9, Mormon Island f/8), and data loggers were engineered by TRLcam (TRLcam.com, Lincoln, Nebraska). Light values, a proxy for sun illumination on the landscape, were calculated in accordance with Harvey [2], using the formula

$$\mathrm{Lv}=\left(\log^{2}\left(\frac{N^{2}}{t}\right)\right)\;+\;\log^{2}\left(\mathrm{ISO}/100\right).$$

Four acoustic recorders (Song Meter SM2+; Wildlife Acoustics, Maynard, Massachusetts, USA) were installed at Rowe Sanctuary, Mormon Island, Beaver Lodge, and Trout Pond and recorded continuously from 10:30 to 15:30 with a sample rate of 16 kHz. These recordings were analyzed by assessing avian vocalization activity and generating acoustic index values. Within each of the 2.5-min phases (pre-eclipse, 95%a, totality, 95%b, post-eclipse), a single observer proficient in regional bird call identification listened to and manually identified the species of all audible vocalizations (songs and calls). To derive acoustic index values, sound recordings were split into 117, 2.5-min intervals from 10:30 to 15:30. We calculated RMS to assess changes in broadband sound levels [4], ACI to measure the acoustic activity of birds and other animals with fast-changing calls [3], and ASA to characterize the acoustic activity of insects and birds (see [5] and [6] for further information on acoustic indices).

## Footnotes

Transparency document Transparency document associated with this article can be found in the online version at https://doi.org/10.1016/j.dib.2018.10.008. For funding details see Brinley Buckley et al. 2018 [1].

Appendix A Supplementary data associated with this article can be found in the online version at https://doi.org/10.1016/j.dib.2018.10.008.

## References

[1] Buckley E. Brinley (2018). Assessing biological and environmental effects of a total solar eclipse with passive multimodal technologies. Ecol. Indic..

[2] P. Harvey, ExifTool: Read, write and edit meta information. Software package available at 〈http://www.sno.phy.queensu.ca/~phil/exiftool〉, 2013.

[3] Pieretti N. (2011). A new methodology to infer the singing activity of an avian community: the Acoustic Complexity Index (ACI). Ecol. Indic..

[4] Pijanowski B. (2011). Soundscape ecology: the science of sound in the landscape. Bioscience.

[5] Sueur J. (2014). Acoustic indices for biodiversity assessment and landscape investigation. Acta Acust. United Acust..

[6] Towsey M. (2014). The use of acoustic indices to determine avian species richness in audio-recordings of the environment. Ecol. Inform..

