# Supplementary material for: Biological and environmental datasets from the August 2017 total solar eclipse
Source: Data Brief. 2018 Oct 5;21:552–5. doi: 10.1016/j.dib.2018.10.008 (PMC6199816; doi:10.1016/j.dib.2018.10.008)
Supplement: Supplementary file 1 — Transparency document. [file mmc1.docx]

**Conflicts of Interest**

The au­thors de­clare that the data was collected in the ab­sence of any com­mer­cial or fi­nan­cial re­la­tion­ships that could be con­strued as a po­ten­tial con­flict of in­ter­est.
